# Personal bias in nutrition advice: A survey of health professionals' recommendations regarding dairy and plant-based dairy alternatives

**DOI:** 10.1016/j.pecinn.2021.100005

**Published:** 2021-11-01

**Authors:** Bridget E. Clark, Lizzy Pope, Emily H. Belarmino

**Affiliations:** a Department of Nutrition and Food Sciences, University of Vermont, Burlington, VT, USA; b Gund Institute for Environment, University of Vermont, Burlington, VT, USA

**Keywords:** Health care professionals, Counseling, Nutrition requirements, Plant-based nutrition, Dairy products

## Abstract

**Objective:**

To examine the association between health professionals' personal dietary behaviors and their professional nutrition recommendations on dairy and plant-based dairy alternatives.

**Methods:**

A cross-sectional survey of 331 U.S. health professionals examined willingness to recommend dairy and/or plant-based dairy alternatives to patients across personal milk preference, and dietary pattern.

**Results:**

Plant-based milk preference (OR 4.52; *p* < 0.001) and following a vegetarian dietary pattern (OR 1.91; *p* = 0.019) were associated with greater odds of recommending plant-based dairy alternatives to patients. Plant-based milk preference (OR 0.16; *p* < 0.001), following a vegetarian dietary pattern (OR 0.45; *p* = 0.009), and considering one's diet to be “plant-based” (OR 0.41; *p* = 0.005) were associated with lessor odds of recommending dairy to patients. Dietetics professionals were more likely than all other health professionals to recommend both dairy and plant-based dairy alternatives to patients.

**Conclusion:**

Health professionals' nutrition recommendations may reflect their personal nutrition choices. Improved nutrition training, focusing on evidence-based recommendations, reducing personal bias in practice, and routinely including registered dietitians on interprofessional healthcare teams may improve the quality of nutrition advice given to U.S. consumers.

**Innovation:**

This paper is the first to examine if health professionals' personal health behaviors are associated with their health advice on dairy and/or plant-based dairy alternatives.

## Highlights

• Health providers' milk product recommendations reflect personal diet preferences.
• Dietetics professionals are more willing to recommend dairy and dairy alternatives.
• Improved nutrition counseling training for providers may help reduce personal bias.
• Registered dietitians represent key members of interprofessional healthcare teams.

## 1 Introduction

Americans consider health professionals among the most trustworthy sources of health and nutrition information [1,2]. Health professionals are often called upon to advise patients on dietary choices, and are expected to provide sound nutrition recommendations using the most up to date evidence [3,4]. Unfortunately, many health degree programs other than dietetics programs lack adequate nutrition education [3,[5], [6], [7]], making it difficult for health providers outside of registered dietitian nutritionists (RDN) to accurately and confidently provide nutrition advice [6]. Prior research also shows that health professionals' personal health behaviors may influence the health advice they give to patients [[8], [9], [10], [11], [12]], and evidence suggests they are more confident in counseling patients on health and nutrition behaviors they practice themselves [13]. Work examining this relationship has found that physical activity habits among health professionals and health professional degree students are likely to influence how much physical activity advice they provide [[9], [10], [11]]. Likewise, in a study of U.S. female physicians from the Woman Physicians' Health Study, those who were more concerned with their own eating habits or identified as a vegetarian were more likely to advise patients on nutrition [12]. A study of Canadian physicians found those who consumed more fruits and vegetables advised patients on nutrition more often [9].

Health professionals may be asked by patients for advice on diets that consist mainly of plant-based (PB) foods. In recent years, many Americans have reported reducing their intake of animal-source foods [14,15]. National and international reports have begun to highlight evidence showing the potential long-term health benefits of diets that emphasize vegetables, fruits, and whole grains, and limit intake of animal-source foods [16,17]. Growing evidence demonstrates that PB dietary patterns are protective against multiple non-communicable diseases, including cardiovascular disease [[18], [19], [20]], type 2 diabetes [18,19], obesity [18], and certain cancers [18,19].

As many Americans reduce their animal product intake, sales of substitute products for popular animal-based foods has increased [21]. Among the most popular are dairy alternatives, including PB milk, cheese, and yogurt products [21]. PB dairy alternatives can be useful for individuals who have an allergy or intolerance to dairy [22], or who choose not to consume dairy for ethical reasons such as concerns for the environment or animal welfare [14]. Many consumers also choose dairy alternatives due to perceived health benefits [14,23]. However, evidence of health benefits from omitting dairy from the diet is inconsistent [24]. Some research shows that dairy can have positive health impacts, potentially reducing one's risk of type 2 diabetes and certain cancers [24]. A diet of predominately plant foods may be protective against some chronic diseases [18], but the complete exclusion of a certain food groups may not be, and may lead to risk of nutrient inadequacies [19,20]. Because PB dairy alternatives do not have equal nutrient content to dairy, especially in terms of protein and micronutrients [23,25,26], consumers who choose these products must ensure they are not missing these key nutrients in their diet.

It is important to assess how health professionals advise patients on dairy and PB dairy alternatives, and if they provide unbiased recommendations based on the evidence of health outcomes associated with intake of these products. Accurate nutrition guidance from healthcare providers can positively impact patient dietary behavior [27]. No research to date has examined if a relationship exists between health professionals' personal dietary preferences related to PB diets and their recommendations on dairy and dairy alternative products. The objectives of this study were to examine health professionals' dietary preferences, which milk products (dairy and/or PB) they would recommend to patients and why, and if their personal preferences are associated with their professional advice on either product. We hypothesized that health professionals who follow animal product-reducing or PB dietary patterns or prefer PB milk to dairy milk would be more likely to recommend PB dairy alternatives and less likely to recommend dairy products to patients.

## 2 Methods

We designed a web-based (Qualtrics, Provo, UT) survey to measure health professionals' personal dietary preferences, and their recommendations on dairy products and PB dairy alternatives. To be eligible, individuals had to be age 18 years or older; live in the U.S. since at least January 2020; and currently be a dentist, dental hygienist, doctor of osteopathy, licensed practical nurse, medical doctor, nurse practitioner, physician assistant, RDN, registered nurse, or a student currently enrolled in a degree program for one of those professions holding Junior undergraduate standing or above. If a respondent did not meet all of the above criteria, they were notified that they were ineligible and thanked for their time. These specific categories of health professionals were targeted as likely to have the most direct contact and opportunity to provide nutrition advice to patients. Our survey instrument was designed with input from nutrition and public health researchers with different professional expertise and dietary habits.

The survey asked respondents' age, gender identity, race, ethnicity, state of residence, and type of health profession or professional degree program. To measure health professionals' recommendations on dairy and PB alternatives, we included a 4-item instrument modified from two previous studies [28,29], which asked if respondents would recommend dairy foods to a patient and if they would recommend PB dairy alternatives to a patient. Response options for both questions were yes, no, and unsure. If respondents answered affirmatively to either question, they were asked which purpose(s) would lead them to recommend that product: maintenance of good health; prevention of chronic disease; treatment of chronic disease; nutrient deficiency; intolerance or allergy; or other with a space to elaborate.

We used a series of questions to measure health professionals' dietary patterns. First, an instrument adapted from the National Health and Nutrition Examination Survey (NHANES) Dietary Screener Questionnaire (DSQ) [30,31] asked respondents the major type of milk they consume, and the frequency they consume it. Five response options were provided: from cows, from goats or sheep, PB milk, other, or I don't drink milk. Those who responded other could write in the type of milk consumed. We then asked which type of dietary pattern respondents most closely identify with: vegan (not consuming any animal products); vegetarian (not consuming any meat or fish); semi-vegetarian (consuming red meat, poultry or fish no more than once a week); pesco-vegetarian (consuming no meat but fish); omnivorous (eating meat or fish almost every day); or other with a space to write-in their dietary pattern [32]. Lastly, we asked respondents whether they consider their diet to be “plant-based” to which they could respond affirmatively or negatively.

To reduce bias that could be introduced through utilizing a single method of non-probability convenience sampling, we used three methods to recruit respondents: (1) paid digital ads via Facebook to reach a national sample; (2) targeted postings to relevant national LinkedIn and Facebook group pages; and (3) postings on academic, professional, and community listservs in Vermont as well as nationally. The survey was open from November 19, 2020 until February 4, 2021 and 417 people completed at least part of the survey. For this analysis, we excluded 86 respondents who did not answer both recommendation questions, resulting in a final sample of 331. Respondents were entered in a gift card drawing. This study was determined to be exempt by the Institutional Review Board at the University of Vermont.

We analyzed survey data with IBM SPSS Statistics (version 27). During analysis, variables for race and ethnicity were merged and recoded to a binary variable “Black, Indigenous, or person of color (BIPOC)” and “not BIPOC”. State of residence was recoded to a binary variable that indicated whether it was a “dairy state”. U.S. dairy states were determined based on being among the 10 states with the highest percentage of total farm sales coming from milk sales in 2017 (VT, NM, NY, WI, ID, NH, PA, AZ, MI, and ME) [33]. For milk product preference, respondents who reported other and could not be reclassified or reported not drinking milk were excluded from analyses that included this variable, as an aim of this study was to examine preference for dairy versus PB alternative products. Dietary pattern was recoded to a binary omnivorous/vegetarian variable, with vegetarians representing all respondents who reported a dietary pattern that reduced meat intake. Respondents who selected other as their dietary pattern but described a diet that fit into one of the specified diets were reclassified to that category. Only two respondents could not be reclassified and were excluded from analyses that included this variable.

We generated univariate descriptive statistics for all variables. We used a series of six logistic regression models to calculate odds ratios. We examined whether milk preference, dietary pattern, or considering one's diet to be PB was associated with whether the respondent would recommend (1) dairy foods and (2) PB dairy substitutes. For the models, respondents who selected no or unsure regarding if they would recommend dairy and/or dairy alternatives were combined and compared to respondents who selected yes, they would recommend dairy and/or dairy alternatives. Following the construction of unadjusted models, we adjusted the models for age, whether or not the respondent lived in a dairy state, and health professional type (dietetics professional or student versus other type of health professional or student). Gender identity and race/ethnicity were excluded from the models due to the low number of participants who identified with a gender other than female or with a race other than non-Hispanic white. Health professional type was added to the models because a separate analysis with this dataset found perceptions of dairy and PB alternatives to differ between dietetics professionals/students and other types of health professionals/students [34]. Although nurses made up a large portion of our final sample, we were interested in examining differences in health professionals with and without formal nutrition training, and we therefore grouped nurses with all other non-dietetics professionals. Given the limited scope of practice of licensed practical nurses, we ran a sensitivity analysis excluding these respondents. We found no changes in the significance of the results, and licensed practical nurses were therefore included in our final sample. We used listwise deletion to handle missing data. Tests were statistically significant if *p* < 0.05.

## 3 Results

Most survey respondents were female (91.5%), non-Hispanic white (87.5%), and under age 55 (82.2%) (Table 1). About half (46%) were from a dairy state, reflecting the additional recruitment conducted in Vermont. The sample consisted of 276 practicing health professionals and 55 students hereafter collectively referred to as “health professionals”. RDNs and dietetics students made up 45.3% of the sample, nurses and nursing students made up 39.3% of the sample, and other health professionals or students made up the remainder.

**Table 1:** Characteristics of survey respondents.

Variable		n	%
Age			
18–34		146	44.1
35–54		126	38.1
Over 55		59	17.8
Female		290	91.5
Non-Hispanic white a		266	87.5
Location			
Dairy state		146	46.3
Other state		169	53.7
Health profession			
Dietetics professional or student		150	45.3
Nursing professional or student		130	39.3
Other health professional or student		51	15.4
Would recommend dairy foods to a patient			
Yes		267	80.7
No		24	7.3
Unsure		40	12.1
Would recommend PB dairy alternatives to a patient			
Yes		237	71.6
No		29	8.8
Unsure		65	19.6
Milk product preference			
Dairy milk		145	51.6
Plant-based milk		136	48.4
Frequency of milk consumption			
<Once per day		161	57.7
≥Once per day		118	42.3
Dietary pattern			
Omnivore		193	59.6
Vegetarian		131	40.4
Follow a plant-based diet			
Yes		132	41.5
No		186	58.5

*Note*. *N* = 331. Totals for gender identity (*n* = 317), race/ethnicity (*n* = 304), location (*n* = 315), milk preference (*n* = 281), frequency of milk consumption (*n* = 279), dietary pattern (*n* = 324), and following a PB diet (*n* = 318) are smaller because response to these questions was optional.

a The small number of Black, Indigenous, and people of color (BIPOC) respondents inhibited the disaggregation of analyses by race/ethnicity.

**Fig. 1:**
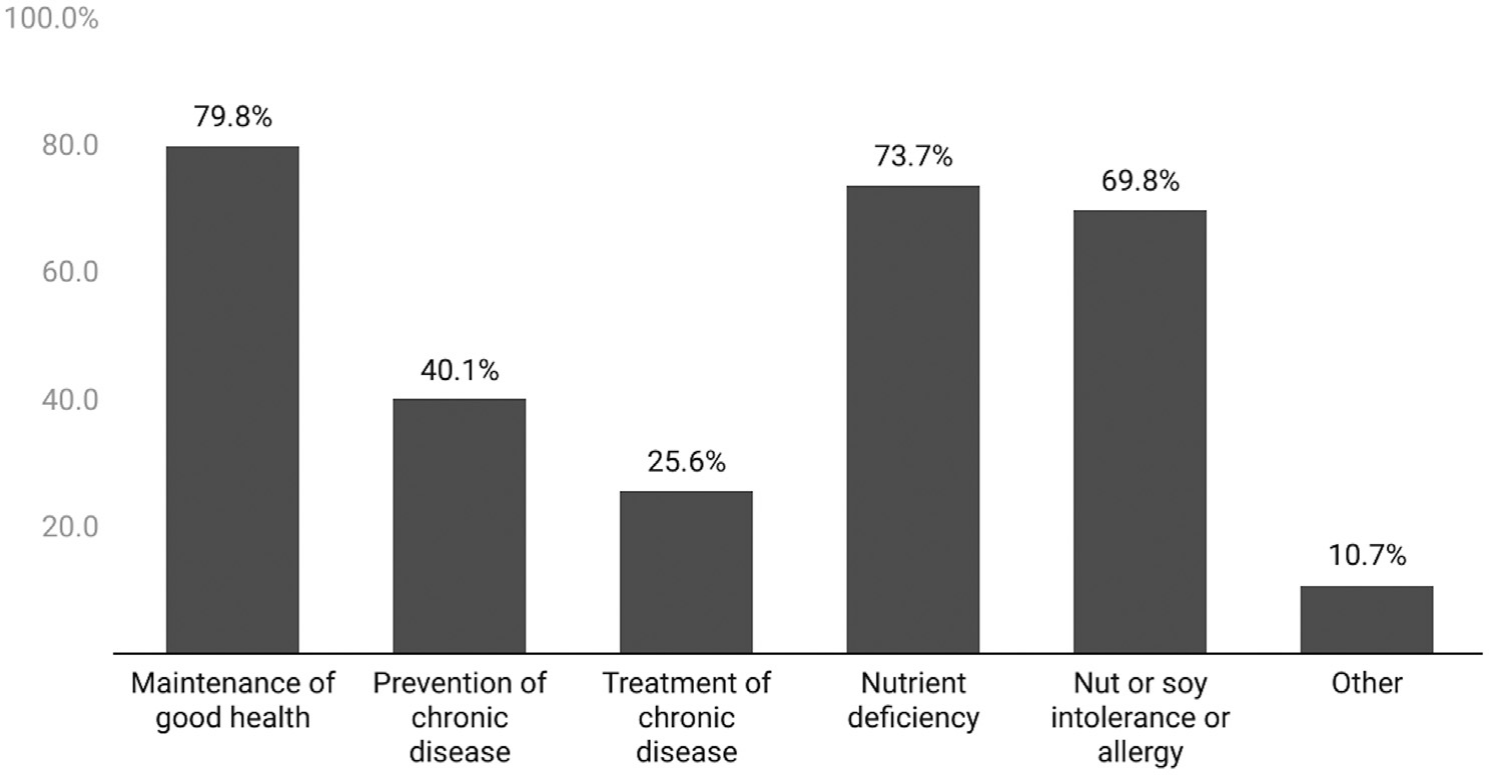
Percentage of respondents who would recommend dairy foods to a patient for each purpose. Question was only asked to respondents who selected ‘yes’ they would recommend dairy foods to a patient (*n* = 262).

**Fig. 2:**
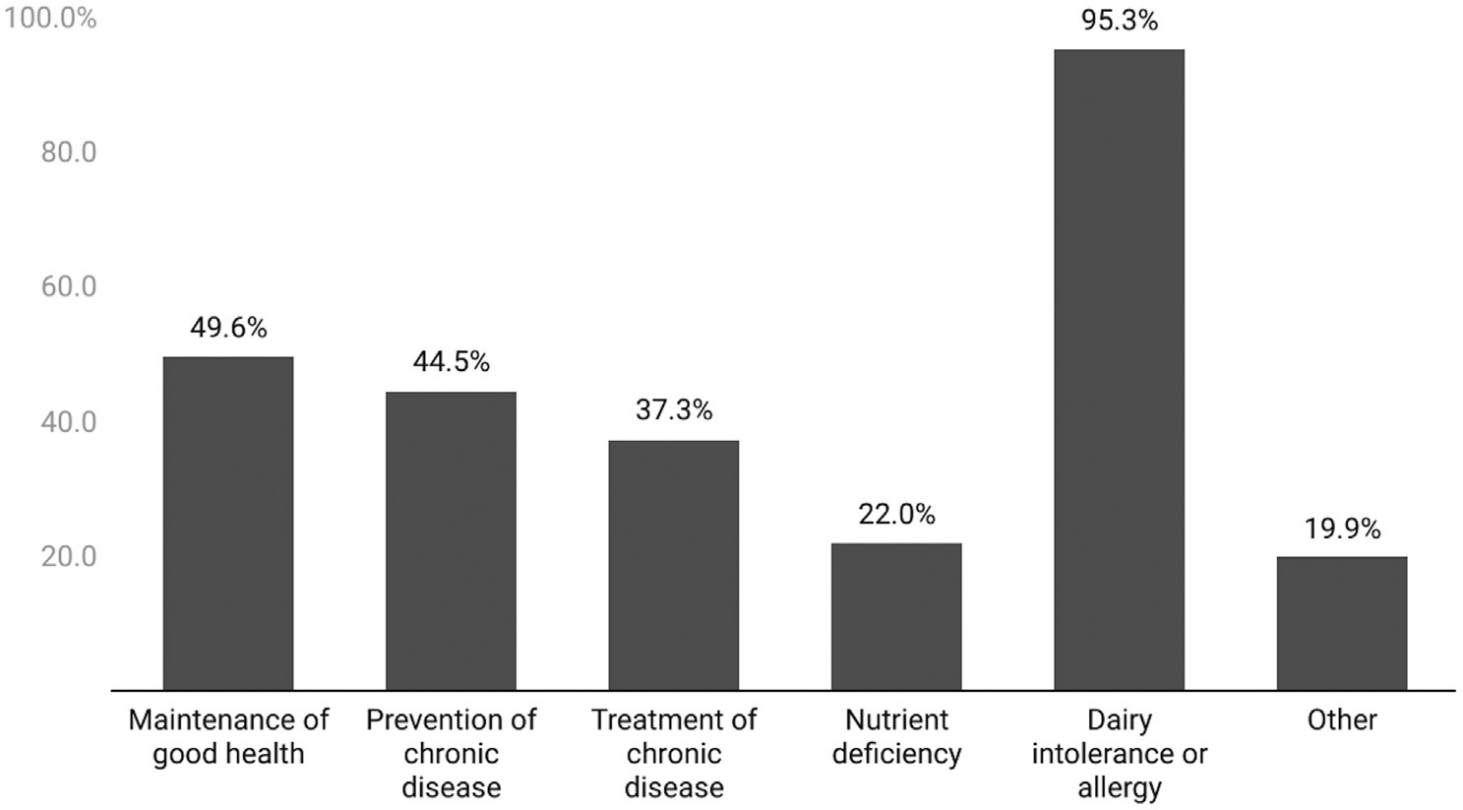
Percentage of respondents who would recommend PB dairy alternatives to a patient for each purpose. Question was only asked to respondents who selected ‘yes’ they would recommend PB dairy alternatives to a patient (*n* = 236).

The majority of health professionals would recommend both dairy (80.7%) and PB dairy alternatives (71.6%) to their patients (Table 1). Top reasons health professionals would recommend dairy foods to a patient included maintenance of good health (79.8%), a nutrient deficiency (73.7%), or a nut or soy allergy or intolerance (69.8%) (Fig. 1). Dairy allergy or intolerance far outweighed all other reasons health professionals would recommend PB dairy alternatives to a patient, with almost all (95.3%) citing this reason (Fig. 2). Of the 19.9% who stated “other” for why they would recommend dairy alternatives, almost half said the patient's personal preference.

Almost half of respondents (48.4%) consume PB milk alternatives over dairy milk (Table 1). A little over half (57.7%) drink dairy or PB milk infrequently (less than once a day), and 42.3% drink milk frequently (once a day or more). About 60% of respondents reported following an omnivorous dietary pattern and about 40% reported following some form of a vegetarian diet. Likewise, about 40% of respondents considered their diet to be PB.

In unadjusted regression models, preference for PB milk (OR 0.14; 95% CI 0.06 to 0.30, *p* < 0.001) and identifying as vegetarian (OR 0.53; 95% CI 0.30 to 0.92, *p* = 0.025) were significantly associated with lessor odds of recommending dairy foods to patients. Preference for PB milk (OR 3.76; 95% CI 2.09 to 6.79, *p* < 0.001), identifying as a vegetarian (OR 2.05; 95% CI 1.22 to 3.46, *p* *=* 0.007), and following a PB diet (OR 1.90; 95% CI 1.14 to 3.19, *p* = 0.015) were significantly associated with greater odds of recommending dairy alternatives to patients (Table 2).

**Table 2:** Unadjusted regression models examining whether respondents would recommend dairy and PB dairy alternative products to their patients from personal preferences and dietary pattern.

Model	Variable	Would recommend dairy products to a patient				
		b	SE	OR	CI	*p* value
1	Milk preference (ref = milk from animals)					
	Plant-based milk	−1.98	0.39	0.14	0.06–0.30	**< 0.001**
2	Dietary pattern (ref = omnivore)					
	Vegetarian	−0.64	0.29	0.53	0.30–0.92	**0.025**
3	Follow a plant-based diet (ref = no)					
	Yes	−0.55	0.29	0.58	0.33–1.02	0.058

		Would recommend dairy alternatives to a patient				
		b	SE	OR	CI	p value

4	Milk preference (ref = milk from animals)					
	Plant-based milk	1.33	0.30	3.76	2.09–6.79	**< 0.001**
5	Dietary pattern (ref = omnivore)					
	Vegetarian	0.72	0.27	2.05	1.22–3.46	**0.007**
6	Follow a plant-based diet (ref = no)					
	Yes	0.64	0.26	1.90	1.14–3.19	**0.015**

*Note*. The logistic regression models presented above used “no or unsure” as the reference category of the dependent variables. Milk preference *n* = 281; Dietary pattern *n* = 324; Follow a PB diet *n* = 318.

In adjusted regression models, dietary preferences and health profession were associated with respondents' willingness to recommend dairy and PB dairy alternatives to patients. In the first adjusted model examining milk preference (Table 3), health professionals who prefer PB milk were significantly less likely to say they would recommend dairy products to a patient (OR 0.16; 95% CI 0.07 to 0.35, *p* < 0.001) and more likely to say they would recommend dairy alternatives to patient (OR 4.52; 95% CI 2.31 to 8.82, *p* < 0.001). Frequent milk product drinkers were significantly less likely to say they would recommend dairy alternatives to a patient (OR 0.47; 95% CI 0.26 to 0.86, *p* = 0.014). As compared to non-dietetics professionals, dietetics professionals were significantly more likely to say they would recommend both dairy (OR 2.85; 95% CI 1.36 to 5.95, *p* = 0.005) and dairy alternatives (OR 2.79; 95% CI 1.49 to 5.25, *p* = 0.001) to a patient.

**Table 3:** Adjusted regression examining if respondents who prefer PB milk over dairy would recommend dairy and PB dairy alternative products to their patients (*n* = 272).

Variable		Would recommend dairy products to a patient						Would recommend dairy alternatives to a patient				
		b	SE	OR	CI	p value		b	SE	OR	CI	p value
Age (ref = 18–34)												
35–54		−0.16	0.40	0.85	0.39–1.86	0.684		0.35	0.35	1.41	0.71–2.82	0.328
Over 55		−0.80	0.52	0.92	0.33–2.58	0.878		0.03	0.42	1.03	0.46–2.32	0.948
Location (ref = Not Dairy State)												
Dairy state		0.33	0.36	1.39	0.68–2.81	0.365		−0.24	0.30	0.79	0.44–1.43	0.436
Health professional type (ref = non-dietetics professional)												
Dietetics professional		1.05	0.38	2.85	1.36–5.95	**0.005**		1.03	0.32	2.79	1.49–5.25	**0.001**
Milk frequency (ref ≤ once per day)												
≥ once per day		−0.01	0.35	0.99	0.50–1.94	0.971		−0.75	0.31	0.47	0.26–0.86	**0.014**
Milk preference (ref = milk from animals)												
Plant-based milk		−1.85	0.41	0.16	0.07–0.35	**< 0.001**		1.51	0.34	4.52	2.31–8.82	**< 0.001**

*Note*. The multivariate logistic regression models presented above used “no or unsure” as the reference category of the dependent variables.

The second adjusted model (Table 4), examining recommendations by dietary pattern, indicated that health professionals who identified as vegetarian were significantly less likely to say they would recommend dairy products to a patient (OR 0.45; 95% CI 0.25 to 0.82, *p* = 0.009), and more likely to say they would recommend dairy alternatives to a patient (OR 1.91; 95% CI 1.11 to 3.27, *p* = 0.019) as compared to health professionals who identified as omnivorous. Dietetics professionals were again significantly more likely to say they would recommend both dairy (OR 1.40; 95% CI 2.03 to 8.03, *p* < 0.001) and dairy alternatives (OR 2.01; 95% CI 1.18 to 3.43, *p* = 0.010) to a patient.

**Table 4:** Adjusted regression examining if respondents who follow a vegetarian diet would recommend dairy and PB dairy alternative products to their patients (*n* = 314).

Variable		Would recommend dairy products to a patient						Would recommend dairy alternatives to a patient				
		b	SE	OR	CI	p value		b	SE	OR	CI	p value
Age (ref = 18–34)												
35–54		0.26	0.34	1.30	0.66–2.55	0.447		0.08	0.30	1.09	0.60–1.96	0.784
Over 55		0.45	0.44	1.57	0.66–3.75	0.311		−0.38	0.35	0.68	0.34–1.36	0.276
Location (ref = Not Dairy State)												
Dairy state		0.54	0.32	1.72	0.92–3.23	0.089		−0.43	0.27	0.65	0.39–1.10	0.108
Health professional type (ref = non-dietetics professional)												
Dietetics professional		1.40	0.35	4.03	2.03–8.03	**< 0.001**		0.70	0.27	2.01	1.18–3.43	**0.010**
Dietary pattern (ref = Omnivore												
Vegetarian		−0.80	0.31	0.45	0.25–0.82	**0.009**		0.64	0.28	1.91	1.11–3.27	**0.019**

*Note*. The multivariate logistic regression models presented above used “no or unsure” as the reference category of the dependent variables.

The third adjusted model (Table 5) found respondents who reported that their diet was PB were significantly less likely to say they would recommend dairy products to a patient (OR 0.41; 95% CI 0.22 to 0.76, *p* = 0.005). Dietetics professionals were significantly more likely to say they would recommend both dairy (OR 4.65; 95% CI 2.29 to 2.45, *p* < 0.001) and dairy alternatives (OR 1.82; 95% CI 1.06 to 3.12, *p* = 0.031) to a patient.

**Table 5:** Adjusted regression examining if respondents who consider their diet to be PB would recommend dairy and PB dairy alternative products to their patients (*n* = 309).

Variable		Would you recommend dairy products to a patient						Would you recommend dairy alternatives to a patient				
		b	SE	OR	CI	p value		b	SE	OR	CI	p value
Age (ref = 18–34)												
35–54		0.17	0.35	1.19	0.60–2.36	0.618		0.14	0.30	1.15	0.63–2.07	0.653
Over 55		0.43	0.44	1.54	0.64–3.67	0.334		−0.42	0.35	0.66	0.33–1.32	0.240
Location (ref = Not Dairy State)												
Dairy state		0.53	0.32	1.70	0.91–3.21	0.099		−0.41	0.27	0.66	0.39–1.11	0.120
Health professional type (ref = non-dietetics professional)												
Dietetics professional		1.54	0.36	4.65	2.29–9.45	**< 0.001**		0.60	0.28	1.82	1.06–3.12	**0.031**
Follow a plant-based diet (ref = No)												
Yes		−0.90	0.32	0.41	0.22–0.76	**0.005**		0.51	0.28	1.67	0.97–2.89	0.067

*Note*. The multivariate logistic regression models presented above used “no or unsure” as the reference category of the dependent variables.

## 4 Discussion and conclusion

### 4.1 Discussion

To our knowledge this is the first paper to explore health professionals' nutrition recommendations on dairy and PB dairy alternatives. Almost half of respondents preferred PB milk over dairy milk, and about 40% identified as vegetarian or described their diet as PB. The majority of health professionals we sampled would recommend both dairy and dairy alternative products to patients. However, health professionals who preferred PB milk or followed PB dietary patterns were more likely to recommend dairy alternatives and less likely to recommend dairy.

Maintenance of good health, nutrient deficiency, and nut or soy allergy or intolerance were top reasons health professionals would recommend dairy to a patient, while dairy allergy or intolerance far exceeded all other reasons for health professionals recommending PB dairy alternatives. Our findings agree with prior research indicating dairy allergy or intolerance is a top reason consumers choose PB alternatives [22]. Aligning with current U.S. dietary recommendations [35], a much greater proportion would recommend dairy (compared to PB alternatives) for maintenance of good health. Only about 1 in 5 health professionals in this study who said they would recommend PB products to patients would do so to combat a nutrient deficiency, and less than 50% said they would recommend dairy or a PB product for prevention and/or treatment of chronic disease. This may indicate that many health professionals don't feel that milk products have a large influence on disease risk. Current literature [24] suggests dairy intake is neither the superfood nor the dietary villain that different stakeholders make it out to be [36,37].

The proportion of health professionals who indicated that they prefer to drink PB milk alternatives (48.4%) is higher than the estimated 23% of U.S. households that consume mostly non-dairy milks, as found in a recent representative study of 995 U.S. households [38]. It is interesting that almost half prefer PB milk, as almost half of the sample came from a dairy state where dairy milk consumption may be expected to be higher. This high proportion is also interesting because most of the sample was made up of non-Hispanic white respondents, and this population is more likely to meet U.S. dairy intake recommendations [39]. About 40% of health professionals identified as following a dietary pattern that reduces animal product intake to some extent, and about 40% consider their diets to be PB. Although only about 3–5% of Americans identify as vegetarian or vegan [40,41], almost 40% report trying to consume more PB foods [40]. Prior work has shown mixed results regarding whether the dietary behaviors of health professionals differ from the general public [7,42,43]. Globally, less than 40% of physicians appear to consume more than two servings of fruits and vegetables per day, although over three-fourths appear to consume meat and dairy daily [7]. Another study found that although U.S. female nurses do not consume the recommended amount of fruits and vegetables per day, their intake is higher than women in the general population [43]. Further research may be required to determine the degree that health professionals and all Americans are fully or partially replacing animal-based foods with plant sources.

Health professionals who personally prefer PB milk or identify as vegetarian were more likely to say they would recommend PB dairy alternatives to patients, and less likely to say they would recommend dairy. Health professionals who consider their diet to be PB were also less likely to say they would recommend dairy. In unadjusted models, health professionals with PB diets were also more likely to recommend PB dairy alternatives, although when models were adjusted, this association was no longer significant. Our findings agree with the literature that personal nutrition behaviors are associated with health professionals' nutrition recommendations [9,12]. Although most respondents did report that they would recommend both dairy and PB dairy alternatives to patients, our findings suggest bias in nutrition advising. Whether dairy or a PB dairy alternative is appropriate for a given patient often depends on that patient's individual dietary needs and preferences [14,22]. While the associations between dairy intake and some health outcomes remain unclear [24], dairy is a top food source of many nutrients of concern [35], and advising a patient to choose a PB alternative in place of dairy if there is no personal or health-related need could lead to nutrition risks [19,20]. The nutrition education provided in many health degree programs remains limited [3,[5], [6], [7]]. The greater propensity of RDNs to recommend either product as compared to other health professionals, suggests that their specialized training in nutrition and nutrition counseling may buffer some of the influence of personal preferences. Previous work has highlighted a need to increase interprofessional teamwork between different health professionals [3]. Given the high rates of diet-related non-communicable diseases in the U.S. [44] and the high demand for nutrition information by consumers [45], RDNs represent critical members of interprofessional healthcare teams.

This study has several limitations. First, the relatively small sample size did not allow for certain statistical comparisons, such as the milk product recommendations between vegetarians, vegans, and other meat-reducing dietary patterns separately. However, this may be adequate, given that dietary pattern reporting is often imprecise [46]. Second, few respondents reported that they would not recommend dairy or PB alternatives to their patient, hindering our ability to examine factors that associated with reluctance to recommend these products. Third, health professionals' dietary patterns and preferences were based on self-reported data, which are subject to response bias [47]. What was defined as a PB diet was also left to the respondents' interpretation. Future research on this topic may consider more robust or objective measures of dietary patterns, such as dietary recalls or biomarkers. Fourth, the majority of the sample was female and non-Hispanic white, and nearly 85% were dietetics or nursing professionals. This reflects the additional recruitment activities targeted towards RDNs and nurses, and the extended recruitment in Vermont. Over 90% of RDNs and nurses in the U.S. are female [48,49] and less than 10% of the population of Vermont identify with a race or ethnicity other than non-Hispanic white [50]. The use of an online platform for our survey may have limited the range of health professionals' perspectives we were able to capture. However, 85% of U.S. households now have internet access [51]. Facebook was also a primary means of survey dissemination. About seven in ten U.S. adults use Facebook [52], and most health professionals use social media [53,54,55]. Most prior research examining associations between personal beliefs and behaviors and professional counseling practices has focused on physicians and medical students [8,9,12]. This sample allowed us to examine counseling bias among other types of health professionals.

### 4.2 Innovation

As the consumption of PB dairy alternatives increases in the U.S. [21], it is important to understand if health professionals are providing accurate and unbiased nutrition advice about these products to their patients. While prior work has examined the relationship between health professionals' personal health behaviors and their professional health advice regarding behaviors such as physical activity and fruit and vegetable intake [[8], [9], [10], [11], [12]], this work is innovative as it is the first to examine if health professionals' personal health behaviors, specifically dietary preferences for PB versus dairy milk and/or dietary pattern, are associated with their milk product advice. Additionally, prior work on health behaviors and health advice has not included RDNs [[8], [9], [10], [11], [12]]. This study also was innovative in that it examined personal bias in nutrition advice among health professionals with and without formal dietetics training, which has not been compared previously.

### 4.3 Conclusion

Our findings indicate that the personal milk preferences and dietary patterns of U.S. health professionals may be associated with willingness to recommend dairy and PB dairy alternatives to patients. Health professionals are expected to base their nutrition recommendations on up-to-date nutrition literature.

While PB dairy alternative products are important substitutes for individuals who cannot or will not consume dairy, the needs of the patient should drive nutrition recommendations rather than health professionals' personal beliefs. Future work should focus on strategies for improving nutrition training for health professionals. Specifically, training could focus on use of evidence-based nutrition recommendations. Improved access to information on the nutritional characteristics of both dairy and PB alternatives may increase health professionals' knowledge on this topic regardless of their own dietary practices. Innovation is also needed in education models that focus on collaboration between dietetics and other healthcare disciplines, and the benefit of referring patients with nutrition concerns to RDNs. This may help improve the quality of nutrition advice given to U.S. consumers.

## Data availability statement

Data are available from the corresponding author on reasonable request.

## Funding

This work was supported by the 10.13039/100010941University of Vermont Office of the Vice President for Research (OVPR) through an EXPRESS Grant [Project ID 038152]. This sponsor had no role in the design, data collection, data analysis, or interpretation of the work presented. Additional funding for this work was provided by the University of Vermont College of Agriculture and Life Sciences (CALS).

## Ethics approval

This study was approved by the Committee on Human Research in the Behavioral and Social Sciences (CHRBSS), the Institutional Review Board (IRB) of the University of Vermont. A waiver of documentation of consent was approved by the University of Vermont for the survey. Thorough information about the study was given to survey participants in writing.

We confirm all patient/personal identifiers have been removed or disguised so the patient/person(s) described are not identifiable and cannot be identified through the details of the story.

## Declaration of Competing Interest

In 2021, Lizzy Pope received payment for teaching two courses on intuitive eating.
